# The Importance of Prior Sensitivity Analysis in Bayesian Statistics: Demonstrations Using an Interactive Shiny App

**DOI:** 10.3389/fpsyg.2020.608045

**Published:** 2020-11-24

**Authors:** Sarah Depaoli, Sonja D. Winter, Marieke Visser

**Affiliations:** Department of Psychological Sciences, University of California, Merced, Merced, CA, United States

**Keywords:** Bayesian statistics, prior distributions, sensitivity analysis, Shiny App, simulation

## Abstract

The current paper highlights a new, interactive Shiny App that can be used to aid in understanding and teaching the important task of conducting a prior sensitivity analysis when implementing Bayesian estimation methods. In this paper, we discuss the importance of examining prior distributions through a sensitivity analysis. We argue that conducting a prior sensitivity analysis is equally important when so-called diffuse priors are implemented as it is with subjective priors. As a proof of concept, we conducted a small simulation study, which illustrates the impact of priors on final model estimates. The findings from the simulation study highlight the importance of conducting a sensitivity analysis of priors. This concept is further extended through an interactive Shiny App that we developed. The Shiny App allows users to explore the impact of various forms of priors using empirical data. We introduce this Shiny App and thoroughly detail an example using a simple multiple regression model that users at all levels can understand. In this paper, we highlight how to determine the different settings for a prior sensitivity analysis, how to visually and statistically compare results obtained in the sensitivity analysis, and how to display findings and write up disparate results obtained across the sensitivity analysis. The goal is that novice users can follow the process outlined here and work within the interactive Shiny App to gain a deeper understanding of the role of prior distributions and the importance of a sensitivity analysis when implementing Bayesian methods. The intended audience is broad (e.g., undergraduate or graduate students, faculty, and other researchers) and can include those with limited exposure to Bayesian methods or the specific model presented here.

## Introduction

Through a recent systematic review of the literature in the Psychological Sciences, we know that the use of Bayesian methods is on the rise (van de Schoot et al., 2017). However, this review also highlighted an unnerving fact: Many applied users of Bayesian methods are not properly implementing or reporting the techniques. The goal of this paper is to tackle one of the main issues highlighted in this systematic review—namely, examining the impact of prior distributions through a sensitivity analysis. Understanding the impact of priors, and then making subsequent decisions about these priors, is perhaps the trickiest element of implementing Bayesian methods. Many users of Bayesian estimation methods attempt to avoid this issue by using “diffuse” priors, but this is not always a viable approach because some models need informative priors. The impact of priors (whether diffuse or otherwise) is highly dependent on issues related to model complexity and the structure of the data. Our paper focuses on how to examine the impact of prior distributions in a transparent manner.

As a motivating example, we conducted a small simulation study illustrating the impact of different prior specifications on final model results. This simulation study shows the importance of thoroughly examining the impact of priors through a sensitivity analysis. We also developed an interactive web application (i.e., Shiny App) for users to learn more about the impact of priors and the need for a sensitivity analysis in empirical situations. This App allows users to examine the impact of various prior distribution settings on final model results, ensuring that the user is fully aware of the substantive impact of prior selection. Examining the impact of priors is central to whether Bayesian results are viable, completely understood, and properly conveyed. Our Shiny App aids with further illustrating this issue.

## Goals of the Current Paper

The current paper provides readers with a step-by-step way of thinking about Bayesian statistics and the use of priors. Prior distributions turn out to be one of the most important elements of any Bayesian analysis, largely because of how much weight and influence they can carry regarding final model results and substantive conclusions. Our aims are as follows:

1. Present readers with a friendly introduction to Bayesian methods and the use of priors. We aim to keep the paper accessible to people coming from a wide range of statistical backgrounds, as well as from a variety of different fields.
2. Illustrate the fact that examining the impact of priors is an incredibly important task when interpreting final model results in an applied research setting. We use a small simulation study to illustrate this point.
3. Introduce a new, interactive Shiny App that we developed in order to assist in visualizing important elements of a prior sensitivity analysis.
4. Demonstrate the potential impact of priors through an empirical example using the interactive Shiny App and data that we supply, which provides a tool for readers to explore prior impact in a hands-on setting.
5. Present a set of frequently asked questions regarding priors and a prior sensitivity analysis, as well as candid answers to each question.

## Intended Audience and Organization of the Paper

This paper is aimed at novice users of Bayesian methodology. We have designed the paper to benefit students and researchers coming from a wide range of statistical backgrounds. For example, undergraduate students may find the Shiny App useful to experiment with some basics of Bayesian statistics and visualize what different prior settings look like. More advanced graduate students or researchers may find the simulation study as a helpful illustration for capturing the importance of prior sensitivity analyses. In turn, they may also find the application presented in the Shiny App particularly useful to understand the specific impact of priors for the model presented here. The paper and Shiny App have been constructed to benefit students and researchers coming from a wide array of fields within the social and behavioral sciences, and all material to reconstruct the analyses presented here is available online at: https://osf.io/eyd4r/.

The remainder of this paper is organized as follows. The next section highlights the main reasons that one would potentially want to use Bayesian methods in an applied research context. One of the main reasons that we cover in this section is that some researchers may want to incorporate previous knowledge into the estimation process. This is typically done through something called a *prior distribution* (or *prior*), and the section following describes the potential impact of priors. This section is particularly relevant to the Shiny App that we developed, and the issues surrounding priors largely remain at the crux of recognizing when Bayesian methods are misused or inaccurately portrayed.

Next, we present information surrounding the multiple regression model, which is referenced in the subsequent sections. We then present a small simulation study, which is aimed to highlight the impact that different prior settings can have on the accuracy of final model estimates obtained. These results lead into the importance of conducting a prior sensitivity analysis. The following section presents information surrounding our Shiny App, how it works, and how readers can benefit from using it. We highlight how the App can be used to learn more about the important issue of prior sensitivity analysis within Bayesian statistics, and we also provide an interactive platform for readers to gain a deeper understanding of the issues described here. Finally, the paper concludes with a discussion of frequently asked questions regarding prior sensitivity analysis, as well as final thoughts on the importance of transparency within research conducted via the Bayesian estimation framework.

## Why Are Bayesian Methods Useful in Applied Research?

There are many reasons why a researcher may prefer to use Bayesian estimation to traditional, frequentist (e.g., maximum likelihood) estimation. The main reasons for using Bayesian methods are as follows: (1) the models are too “complex” for traditional methods to handle (see e.g., Depaoli, 2013; Kim et al., 2013; Cieciuch et al., 2014; Depaoli and Clifton, 2015; Zondervan-Zwijnenburg et al., 2019), (2) only relatively small sample sizes are available (see e.g., Zhang et al., 2007; Depaoli et al., 2017a; Zondervan-Zwijnenburg et al., 2019), (3) the researcher *wants* to include background information into the estimation process (see e.g., Zondervan-Zwijnenburg et al., 2017), and (4) there is preference for the types of results that Bayesian methods produce (see e.g., Kruschke, 2013). It is important to note that, regardless of the reasons that Bayesian methods were implemented, a sensitivity analysis of priors is always important to include. In the subsequent sections, we discuss this issue of priors to a greater extent.

## What Do We Know About the Impact of Priors?

The Bayesian literature (using simulation and applied data) has uncovered several important findings surrounding the potential impact of prior distributions on final model results. Some of the literature has shown that prior impact is highly dependent on model complexity, and it is incredibly important to fully examine the influence of priors on final model estimates. In this section, we unpack this issue a bit more, highlighting the reasons one might want to examine their priors.

### Priors Can Impact Results (Sometimes in a Big Way!)

One of the reasons why the use of Bayesian methods is considered controversial is the notion that priors can (and do!) impact final model results. What this means in a practical sense is that a researcher can have a very strong opinion about the model parameter values, and this opinion (via the prior) can drive the final model estimates. There are many research scenarios within the Bayesian context where informative (or user-specified) priors have an impact on final model estimates. Some examples include research with models such as the latent growth mixture model (Depaoli et al., 2017b; van de Schoot et al., 2018), the confirmatory factor analytic model (Golay et al., 2013), and logistic regression (Heitjan et al., 2008).

The reverse is true in that the literature has shown that completely diffuse priors can also impact final model results. Although Bayesian theory indicates that large sample sizes can overcome (or swarm) the information in the prior (see e.g., Ghosh and Mukerjee, 1992), some research indicates that diffuse priors can impact final model estimates even with larger sample sizes—sometimes in an adverse manner. Examples of modeling situations where diffuse priors have been shown in simulation to adversely impact final model estimates include probit regression models (Natarajan and McCulloch, 1998), meta-analysis (Lambert et al., 2005), item response theory (Sheng, 2010), structural equation modeling (van Erp et al., 2018)—of which sensitivity analysis guidelines are also provided for structural equation models, latent growth mixture models (Depaoli, 2013), and multilevel structural equation models (Depaoli and Clifton, 2015). In all of these cases, researchers found that diffuse priors had a substantial (negative) impact on the obtained estimates.

Accurate estimates are harder to obtain for some parameters than others. Specifically, more complex models (especially when coupled with smaller sample sizes) can require additional information for certain model parameters in order to supplement flatter likelihoods. For example, in some of our own investigations, variances can be more difficult to estimate than means when the likelihood is relatively flatter (and more peaked for a mean). Models that have many parameters that are difficult-to-estimate may require more informative priors, at least on some model parameters. If a parameter is associated with a flatter likelihood, and diffuse priors are implemented, then there may not be enough information (from the data likelihood or the prior) to produce an accurate estimate. The most common instances where this problem occurs are with more complex models (e.g., mixture models, multilevel models, or latent variable models), but the issue is common enough that the impact of priors should be examined regardless of the informativeness of the prior settings. An important take-away from this should be not to blindly rely on prior settings without understanding their impact, even if they are intended to be diffuse or they are software-defined default priors.

If a prior is used to help incorporate the degree of (un)certainty surrounding a model parameter, then we would expect it to have *some* impact. However, it is really important to understand that impact and account for it when drawing substantive conclusions. Therefore, Bayesian experts often agree that an important, and needed, element of Bayesian estimation is the inclusion of a *sensitivity analysis* of the priors.

## What Is a Sensitivity Analysis of Priors?

A sensitivity analysis allows the researcher to examine the final model results, based on the original (or reference) prior, in relation to results that would be obtained using different priors. Many Bayesian experts (e.g., Muthén and Asparouhov, 2012; Kruschke, 2015) recommend that a sensitivity analysis should always be conducted, and there has even been a checklist developed (Depaoli and van de Schoot, 2017) that aids in how to conduct and interpret such results in a transparent manner. For applied papers implementing a sensitivity analysis of priors, see: Müller (2012),Depaoli et al. (2017a), or van de Schoot et al. (2018).

The process takes place as follows:

1. The researcher predetermines a set of priors to use for model estimation. These priors can be default priors from the statistical software, or they can be user-specified based on previous knowledge of the model parameters (e.g., based on a simple guess, a meta-analysis of prior literature, interviews with content experts, etc.).
2. The model is estimated, and convergence is obtained for all model parameters.
3. The researcher comes up with a set of “competing” priors to examine; we will describe what this set of priors can look like in the examples below. The point here is *not* to alter the original priors. Rather, it is to examine how robust the original results are when the priors are altered, and the model is re-estimated.^1^ It can also be a method used to identify priors that would serve as a poor choice for the model or likelihood—an issue we expand on more in the discussion.
4. Results are obtained for the “competing” priors and then compared with the original results through a series of visual and statistical comparisons.
5. The final model results are written up to reflect the original model results (obtained in Item 1, from the original priors), and the sensitivity analysis results are also presented in order to comment on how robust (or not) the final model results are to different prior settings.

This last point is particularly important. A systematic review of Bayesian statistics in the Psychological Sciences (van de Schoot et al., 2017) unveiled that sensitivity analyses were only reported in 16.2% of the applied studies over the course of 25 years. What this means is that the majority of applied Bayesian papers published in the field did not thoroughly examine the role or impact of priors.

One of the biggest aids for examining the role or impact of priors can be to visually examine the resulting posterior distributions across many different prior settings. We will highlight some important ways to visualize priors and sensitivity analysis results in a subsequent section when introducing our interactive Shiny App.

Visual aids are particularly important here because they can help the researcher to more easily determine: (1) how different or similar the posterior distributions are when different priors are formed, and (2) whether the difference across sets of results (from different prior settings) is *substantively* important. In the end, this latter point is really what matters most. If several sets of priors produce slightly different posterior estimates but the results are substantively comparable, then the results are showing stability (or robustness) across different prior settings. In this case, the researcher can be more confident that the prior setting is not influencing the substantive conclusions in a large way.

One may take these last statements to mean that we are implying the opposite results would be somehow negative or bad. In other words, is it a problem if my sensitivity analysis results show that the resulting posterior changes in substantively meaningful ways when the prior is altered? The answer is NO. There is not necessarily a “problem” here. It is incredibly informative to theory-based research to uncover that results are dependent on the particular theory (i.e., prior) being implemented. This is not a *bad* result at all. It is just one that requires a bit more care when describing. Whatever the results are of the sensitivity analysis (e.g., whether results are stable or not), they should be thoroughly reported in the results and discussion sections of the paper. These findings can be presented in terms of visual depictions of the posteriors from multiple sets of priors, as defined through the sensitivity analysis. Likewise, results can also be presented in statistical form, where percent “bias,” or deviation, is computed for parameter estimates obtained under different prior settings.^2^ Another alternative when working with diffuse priors could be to report the results across a range of diffuse priors as the main analysis. This tactic might facilitate illustrating the uncertainty surrounding the exact prior specification, especially if various diffuse priors provide varying results.

If the priors are shifted only a small amount in the sensitivity analysis and they result in *very* different results, then it would be beneficial to take a closer look at the model code to ensure everything is properly specified. However, small-to-moderate shifts in the substantive conclusions are not a concern and should just be reported along with the findings and subsequently addressed in the discussion section with respect to learning something about the robustness of results under different prior settings.

Note that the original prior settings are not modified during the sensitivity analysis process. Instead, sensitivity analysis results are presented, and they may be used as evidence that priors should be shifted in some way in a future analysis on another dataset. For transparency reasons, it is important to keep the original prior and not change it because of something that was unveiled in the sensitivity analysis. Doing so would be an instance of Bayesian HARKing (hypothesizing after results are known; Kerr, 1998), which is just as questionable as frequentist HARKing.

## Proof of Concept Simulation: Illustrating the Impact of Priors

Next, we present a small simulation study illustrating the impact of different prior settings on final model estimates. Since there is no way to know the true value of a population parameter in application, it is not possible to know how much bias estimates contain unless a simulation study is conducted. This simulation study sets the stage for the importance of examining prior impact in application, a concept that we focus on in the interactive Shiny App presented in the following section.

### The Model

For illustration purposes, we used the multiple regression model, which is a very common model that is found in the applied psychological literature.^3^ In turn, it also acts as a foundation for many other advanced models [e.g., (multilevel) mixed regression models, or latent growth curve models]. These reasons make the multiple regression model a good candidate for demonstration. In addition, we felt this model, even if unfamiliar to the reader, can be conceptually described and understood without having strong background knowledge of the model. Although we limit our discussion to multiple regression, the prior sensitivity analysis principles that we demonstrate can be broadly generalized to other model forms (e.g., growth curve models, confirmatory factor analysis, mixture models).

**FIGURE 1:**
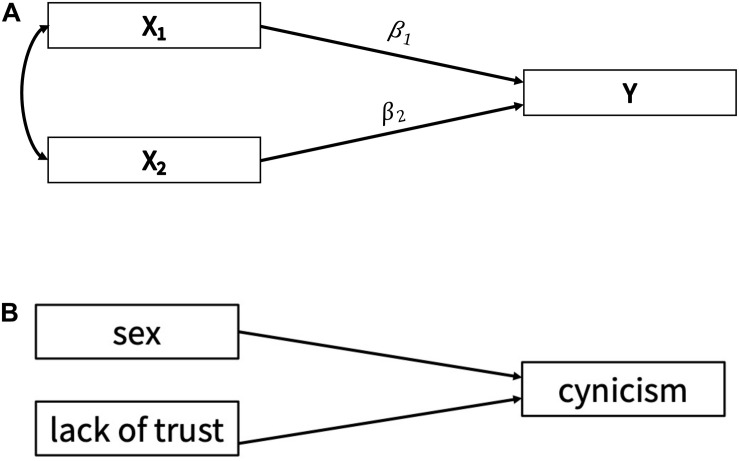
**(A)** Multiple regression model used in simulation study, with a single outcome variable, *Y*, and two predictors, *X*_1_ – *X*_2_. **(B)** Multiple regression model used in the applied example, with an outcome of *Cynicism* and two predictors.

This model has been used in a variety of research settings within the social and behavioral sciences. For example, it has been used to predict academic achievement (Adeyemo, 2007), self-reassurance (Kopala-Sibley et al., 2013), and sleep quality (Luyster et al., 2011). The base of the model includes a single (continuous) outcome variable that is predicted by several different predictor variables; the model can be found in Figure 1A. In this figure, there is a single outcome variable (called “Y”), and two correlated predictors (called “*X*_1_ − *X*_2_”) with regression weights β_1_ − β_2_.

Bayesian methods can be implemented in this modeling context in a relatively simple manner. For a basic form of the model, as seen in Figures 1A,B, a researcher may be particularly interested in placing informative priors on the regression weights (i.e., the directional paths in the figure) that link the predictors to the outcome. In this case, it may mean that the researcher has a particular idea (or theory) about how the variables relate, as well as how strong of a predictor each variable may be in the model.

**FIGURE 2:**
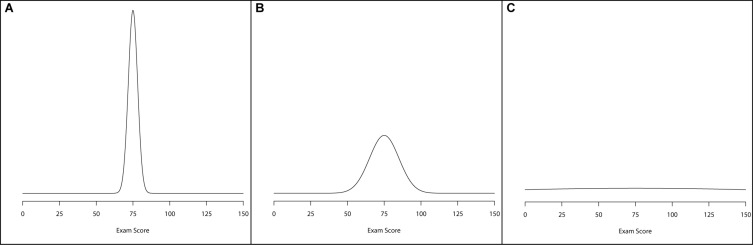
Examples of prior. distributions that are: **(A)** informative, **(B)** weakly informative, and **(C)** diffuse.

Typically, informativeness of a prior is defined by one of three categories: informative, weakly informative, and diffuse. Informative priors are usually conceptualized as priors with a large amount of information surrounding a particular parameter. What this translates to is a large probability mass hovering over a relatively narrowed span of possible values for a parameter to take on. For example, Figure 2A illustrates an informative prior, with narrowed variation surrounding a mean value of 75. A weakly informative prior is one that carries more spread, or variation, than an informative prior. Figure 2B illustrates a weakly informative prior by highlighting a wider distributional spread. Finally, a diffuse prior is one that offers little-to-no information about the parameter value. One way of conceptualizing this prior form is to use a normal prior with a very wide variance, making it effectively flat across a wide range of values. Figure 2C illustrates a diffuse prior setting for the normal distribution. In all three of these plots, the normal prior was centered at 75, but the variance of the priors differed from small (Figure 2A) to large (Figure 2C).^4^

Next, we illustrate how priors can impact final model estimates, even for a model as simple as a multiple regression model. Specifically, we conducted a small simulation study illustrating the effect of different prior settings.

### Simulation Design

The simulation study used a multiple regression model as displayed in Figure 1A. It contained two continuous predictors, a correlation parameter linking these predictors, and a continuous outcome. The population values for these parameters are listed in Table 1. In this simulation, we implemented various sets of priors for the regression coefficients linking the two predictors to the outcome. These prior conditions are listed in Table 1. Overall, there were 11 prior conditions examined per sample size.

**TABLE 1:** Population values and simulation conditions for the multiple regression model.

Population values for simulation	
Parameter	Population value
Means	
*X*_1_	Fixed to 0^1^
*X*_2_	Fixed to 0
Variances	
*X*_1_	Fixed to 1
*X*_2_	Fixed to 1
*Y* Intercept	1
*Y* Resid. Var.	0.5
β_1_	1.0
β_2_	0.5

**Simulation conditions (sample sizes crossed with prior conditions)**	

Sample sizes	Prior conditions^2^
*n* = 25	Informative:
*n* = 100	(1) β_1_ ∼ *N*(0.25, 0.05); β_2_ ∼ *N*(0.125, 0.05)
*n* = 1,000	(2) β_1_ ∼ *N*(0.50, 0.05); β_2_ ∼ *N*(0.250, 0.05)
	(3) β_1_ ∼ *N*(1.00, 0.05); β_2_ ∼ *N*(0.500, 0.05)
	(4) β_1_ ∼ *N*(2.00, 0.05); β_2_ ∼ *N*(1.000, 0.05)
	(5) β_1_ ∼ *N*(3.00, 0.05); β_2_ ∼ *N*(1.500, 0.05)
	Weakly Informative:
	(6) β_1_ ∼ *N*(0.25, 0.1); β_2_ ∼ *N*(0.125, 0.1)
	(7) β_1_ ∼ *N*(0.50, 0.1); β_2_ ∼ *N*(0.250, 0.1)
	(8) β_1_ ∼ *N*(1.00, 0.1); β_2_ ∼ *N*(0.500, 0.1)
	(9) β_1_ ∼ *N*(2.00, 0.1); β_2_ ∼ *N*(1.000, 0.1)
	(10) β_1_ ∼ *N*(3.00, 0.1); β_2_ ∼ *N*(1.500, 0.1)
	Diffuse
	(11) Regression 1 ∼ *N*(0, 10^10^); Slope ∼ *N*(0, 10^10^)

*Y, the continuous outcome in the model. Resid. Var., residual variance. Predictors 1 and 2 (X_1_ and X_2_) were both continuous predictors. β_1_ = Y on X_1._ β_2_ = Y on X_2._^1^The means and variances for the predictors were fixed in the model in order to standardize the predictors. Therefore, estimates are only available for the four remaining parameters. ^2^The remaining priors in the model were default diffuse prior settings as implemented in Mplus.*

Conditions 1–5 specified informative priors on the regression parameters linking each of the predictors to the outcome. These informative priors were not all *correct* in that some of them had inaccurate mean hyperparameter settings for the prior (i.e., the normal prior was not centered on the population value, rather it was shifted away).^5^ Condition 3 is a correct informative prior in that it is centered at the population value and has a relatively narrowed variance. Conditions 1–2 had priors that were shifted downward from the population value, and Conditions 4–5 had priors that were shifted upward.

Conditions 6–10 represented weakly informative priors in that the variance hyperparameter was increased compared to the informative conditions (1–5). The same pattern was exhibited where Condition 8 represented a prior setting with a mean hyperparameter that was accurate to the population value. Conditions 6–7 had mean hyperparameter values that were shifted downward from the truth of the population, and Conditions 9–10 had mean hyperparameters shifted upward.

Finally, Condition 11 represented a diffuse prior, which implemented default settings from M*plus* (Muthén and Muthén, 1998-2017) on the regression parameters. Each of these conditions represented either informative (1–5), weakly informative (6–10), or diffuse priors. Within the informative and weakly informative conditions, we specified (according to the mean hyperparameter) either accurate priors (3 and 8), downward shifted priors (1–2, 6–7), or priors shifted upward from the truth (4–5, 9–10). The goal of these conditions was to highlight the deviation patterns across the sensitivity analysis, with a focus on sensitivity of results to the mean hyperparameter (i.e., accuracy of the mean of the prior) and the variance hyperparameter (i.e., the spread of the prior distribution).

In addition, we also examined the results across three different sample sizes: *n* = 25, 100, and 1000. These sample sizes ranged from relatively small to relatively large, and they were selected to provide information about how priors impact results differently as sample sizes shift.

In all, there were 33 cells in this simulation, and we requested 500 iterations per cell. All analyses were conducted in M*plus* version 8.4 (Muthén and Muthén, 1998-2017) using the Bayesian estimation setting with Gibbs sampling. For simplicity, all cells were set up to have a single chain per parameter, with 5,000 iterations in the chain and the first half discarded as the burn-in (i.e., 2,500 iterations were left to form the estimated posterior). Convergence was monitored with the potential scale reduction factor (PSRF, or R-hat; Gelman and Rubin, 1992a, b), and all chains converged for all cells in the design under a setting 1.01 for the convergence criterion. Another index that can be checked is the effective sample size (ESS), which is directly linked to the degree of dependency (or *autocorrelation*) within the chain. Zitzmann and Hecht (2019) recommend that ESSs over 1,000 are required to ensure that there is enough precision in the chain. Simulation results indicated that, although the post burn-in portions of the chain were only 2,500 iterations, all of the parameters exceeded the minimum of ESS = 1,000 in the cells examined.^6^

### Simulation Findings

Table 2 presents relative percent bias for all model parameters across sample sizes and the 11 prior conditions. Of note, Conditions 3 and 8 represent accurate priors (informative and weakly informative, respectively), and Condition 11 reflects diffuse prior settings. All other priors are either shifted upward or downward, as would be implemented in a sensitivity analysis. Bolded values in the table represent problematic bias levels exceeding ±10% bias.

**TABLE 2:** Model parameter estimate percent bias (MSE) for the simulation study.

Condition	*Y* Intercept	*Y* Resid. Var.	β _1_	β _2_
***n* = 25**				
1	**34.91** (0.0508)	−0.10 (0.2979)	−**40.29** (0.1745)	−**38.50** (0.0474)
2	**21.38** (0.0452)	−0.18 (0.1811)	−**25.66** (0.0774)	−**23.82** (0.0245)
3	**11.90** (0.0403)	−0.34 (0.1239)	−0.05 (0.0115)	1.82 (0.0105)
4	**54.99** (0.0520)	−0.70 (0.5269)	**56.87** (0.3383)	**58.30** (0.0961)
5	**350.76** (0.1468)	−0.68 (14.0520)	**157.51** (2.4857)	**157.86** (0.6300)
6	**22.42** (0.0461)	−0.12 (0.1914)	−**26.50** (0.0919)	−**23.94** (0.0328)
7	**16.66** (0.0434)	−0.18 (0.1513)	−**17.16** (0.0499)	−**14.60** (0.0235)
8	**12.38** (0.0408)	−0.30 (0.1266)	−0.02 (0.0199)	2.48 (0.0184)
9	**30.36** (0.0453)	−0.62 (0.2582)	**36.66** (0.1601)	**38.82** (0.0580)
10	**159.46** (0.0866)	−1.08 (3.3256)	**104.37** (1.1327)	**105.58** (0.3039)
11	**15.37** (0.0426)	−0.20 (0.1451)	0.03 (0.0469)	4.08 (0.0447)
***n* = 100**				
1	4.44 (0.0106)	0.00 (0.0211)	−**13.59** (0.0265)	−**12.58** (0.0111)
2	3.16 (0.0104)	0.02 (0.0194)	−9.06 (0.0160)	−8.06 (0.0086)
3	2.17 (0.0103)	0.04 (0.0183)	−0.26 (0.0076)	0.70 (0.0068)
4	6.37 (0.0107)	0.08 (0.0245)	**17.76** (0.0395)	**18.64** (0.0155)
5	**23.57** (0.0123)	0.14 (0.0908)	**40.44** (0.1742)	**41.22** (0.0502)
6	2.98 (0.0105)	0.00 (0.0192)	−7.55 (0.0151)	−6.46 (0.0094)
7	2.60 (0.0104)	0.00 (0.0187)	−5.12 (0.0119)	−4.04 (0.0087)
8	2.30 (0.0104)	0.02 (0.0184)	−0.30 (0.0091)	0.76 (0.0082)
9	3.52 (0.0105)	0.04 (0.0199)	9.40 (0.0180)	**10.42** (0.0108)
10	7.52 (0.0108)	0.06 (0.0272)	**19.79** (0.0490)	**20.76** (0.0191)
11	2.49 (0.0104)	−0.02 (0.0186)	−0.36 (0.0112)	0.84 (0.0100)
***n* = 1000**				
1	0.27 (0.0010)	−0.36 (0.0020)	−1.33 (0.0012)	−1.56 (0.0010)
2	0.26 (0.0010)	−0.36 (0.0020)	−0.83 (0.0010)	−1.08 (0.0009)
3	0.25 (0.0010)	−0.36 (0.0020)	0.16 (0.0010)	−0.08 (0.0009)
4	0.30 (0.0010)	−0.36 (0.0020)	2.14 (0.0014)	1.90 (0.0010)
5	0.44 (0.0010)	−0.36 (0.0021)	4.12 (0.0027)	3.88 (0.0013)
6	0.26 (0.0010)	−0.36 (0.0020)	−0.59 (0.0010)	−0.84 (0.0010)
7	0.25 (0.0010)	−0.36 (0.0020)	−0.34 (0.0010)	−0.58 (0.0009)
8	0.25 (0.0010)	−0.36 (0.0020)	0.16 (0.0010)	−0.08 (0.0009)
9	0.26 (0.0010)	−0.36 (0.0020)	1.16 (0.0011)	0.92 (0.0010)
10	0.30 (0.0010)	−0.36 (0.0020)	2.16 (0.0014)	1.92 (0.0010)
11	0.25 (0.0010)	−0.36 (0.0020)	0.16 (0.0010)	−0.08 (0.0010)

*Prior conditions (column 1) are described in Table 1. MSE, mean square error, which captures a measure of variability accompanied by bias for the simulation estimates. It can be used as a measure that reflects efficiency and accuracy in the simulation results. Bolded values represent percent bias exceeding 10%. Percent bias = [(estimate − population value)/population value] ^∗^ 100. β_1_ = Y on X_1_. β_2_ = Y on X_2_.*

The most notable finding is how the impact of the priors diminishes as sample size increases. By the time sample size was increased to *n* = 1,000 (which would be rather large for such a simple model), the prior settings had virtually no impact on findings. However, under the smaller sample sizes, and especially *n* = 25, we can see a noticeable impact on results. As the priors were shifted for the regression parameters, bias increased in magnitude. This effect occurred in the more extreme conditions even when *n* = 100, which is not an unreasonable sample size to expect in applied research implementing such a model.

Mean square errors (MSEs) are also presented in Table 2 for each parameter. MSE represents a measure of variability and bias. Notice that MSE values are quite high for *n* = 25, but they decrease to a relatively smaller range as sample sizes are increased to *n* = 100 and beyond. This pattern indicates that sample size has a large role in the efficiency and accuracy of the estimates, as measured through the MSE. In addition, MSEs are much larger for priors that are centered away from the population value.

The practical implication of this simulation highlighted that priors can impact findings (which is indisputable in the Bayesian literature), even when sample sizes are what we might consider to be reasonable. This fact makes sensitivity analyses indispensable when examining the impact of priors on final model results, and examining prior impact is especially important under smaller sample sizes. In practice, researchers do not *know* if subjective priors are accurate to the truth. We argue that researchers should assume that priors have at least some degree of inaccuracy, and they should assess the impact of priors on final model estimates keeping this notion in mind. The only way to truly examine the impact of the prior when working with empirical data is through a sensitivity analysis.

This proof of concept simulation provides a foundation for the Shiny App, which uses empirical data to further illustrate the importance of conducting a sensitivity analysis. In the next section, we present the Shiny App as an educational tool for highlighting the impact of prior settings. A main focus of the App is to illustrate the process of conducting a sensitivity analysis, as well as the type of results that should be examined and reported when disseminating the analysis findings. Specifically, we describe how one would manipulate the settings to examine the impact of priors on final model results. The Shiny App can be used to gain a deeper understanding of the impact of priors, as well as understand the different elements that are needed to properly display sensitivity analysis results.

## Sensitivity Analysis in Action: An Interactive App

To illustrate the importance and use of prior sensitivity analysis, we created an interactive application using rstan (Stan Development Team, 2020), Shiny (Chang et al., 2020), and RStudio (R Core Team, 2020; RStudio Team, 2020). The App can be accessed online at https://ucmquantpsych.shinyapps.io/sensitivityanalysis/. Alternatively, it is available for download on the Open Science Framework^7^. To run the App on your personal computer, open the *ui.R* and *server.R* files in RStudio and press the “Run App” link in the top-righth and corner of the R Script section of the RStudio window. For more information about Shiny Apps, we refer to RStudio Team (2020).

Our App consists of seven different tabs, with each containing information that will help a user understand how to assess the substantive impact of prior selection. When the App is first loaded, it defaults to the first tab. This tab introduces the App, goes over the main steps of a sensitivity analysis, and describes the other tabs of the App. Within the second tab, a fictional researcher and their study are introduced. Specifically, a researcher has collected a sample of 100 participants to examine whether an individual’s sex or lack of trust in others predicts the individual’s cynicism (see Figure 1B for an illustration of the model). The tab discusses the prior distributions specified by the researcher. While most prior distributions are relatively diffuse (i.e., flat), the researcher specifies an informative prior for the regression effect of cynicism on lack of trust. The remainder of the tab focuses on an evaluation of the posterior results of the original analysis, using trace plots, posterior density plots and histograms, and relevant summary statistics [e.g., posterior mean, SD, 90% highest posterior density interval (HPD interval)].

In the next four tabs, users can specify alternative prior distributions for each parameter in the model: the intercept of cynicism (third tab), the regression effect of cynicism on sex (fourth tab), the effect of cynicism on lack of trust (fifth tab), and the residual variance of cynicism (sixth tab). Within these tabs, the priors for the other parameters are held constant. The user can specify and assess the impact of two alternative prior distributions at a time. Each time a new set of priors is specified, additional analyses are run using the rstan package.^8^ The tabs include visual and numerical comparisons that can help assess the impact of the specified prior distributions.

In the seventh tab, users can combine the alternative prior specifications from the previous four tabs to investigate the combined influence of alternative priors on the posterior estimates. Use of the App will be demonstrated in the next section.

### Sensitivity Analysis Process

In this section, we will use the Shiny App to execute and report a sensitivity analysis. The first step is to identify the original (comparison) priors that are to be implemented in the investigation. Then the researcher would carry out a sensitivity analysis to examine the robustness of results under different prior specifications. The researcher would specify alternative priors to explore through the sensitivity analysis process. In this section, we will highlight a sensitivity analysis for two parameters in the model, both of which can be captured through the normal distribution. Although there are many distributional forms that priors can take on, the normal distribution is an effective place to start since it is so visually illustrative of the different forms the normal prior can adopt. As a result, we discuss sensitivity analysis in terms of this prior, but it is important to recognize the issues and processes that we highlight can generalize to other distributional forms. For example, a sensitivity analysis for the residual variance of cynicism can also be examined through the App. The prior for this parameter follows an inverse gamma (IG) distribution. In addition to the conjugate distributions (i.e., the prior and posterior distribution are in the same probability distribution family) used in the App, it is also possible to examine non-conjugate priors (e.g., a reference prior). We did not include alternative, non-conjugate, distributions in our App, as we felt it would distract from its main pedagogical purpose. For more information on non-conjugate priors, see Gelman et al. (2014, p. 36+). An example of a write-up for the prior sensitivity analysis can be seen in the Appendix.

### Specifying Priors on Certain Model Parameters

Priors are specified on all parameters of a model. In this example, we will focus on just two model parameters to illustrate the process of sensitivity analysis. These two parameters are the regression coefficients linking the two predictors to the outcome of *Cynicism*. A separate sensitivity analysis can be conducted on each parameter, and another analysis examines the combined specification of the priors. This latter combined analysis helps to pinpoint the combined impact of a set of alternative priors on all parameters in the model.

#### Parameter 1: *Cynicism* on *Sex*

The researcher can examine competing prior specifications for the effect of *Cynicism* on *Sex*. For example, if the experts originally assumed that there was no *Sex* effect, then a prior such as *N*(0,10) could be specified, where the bulk of the distribution is centered around zero. Notice that this prior is weakly informative surrounding zero (i.e., it still contains ample spread about the mean, as opposed to being strictly informative). For the sake of this example, this prior setting can be viewed as the original prior in the analysis.

Alternative prior specifications can be examined through the sensitivity analysis, in order to examine the impact of different priors (perhaps reflecting different substantive theories) on final model results. For example, another theory could state that men (coded as 1) possess higher levels of cynicism than women, suggesting a positive effect. An informative prior centered around a positive value can be explored to examine this prior belief: e.g., *N*(5, 5). Alternatively, there may be competing research that indicates that men possess lower levels of cynicism than women, suggesting a negative effect. An informative prior centered around a negative value can be explored to examine the impact of this prior belief on the posterior results: *N*(−10, 5). These prior settings would result in an original prior and two alternative specifications such that:

• Original = *N*(0, 10)
• Alternative 1 = *N*(5, 5)
• Alternative 2 = *N*(−10, 5).

**FIGURE 3:**
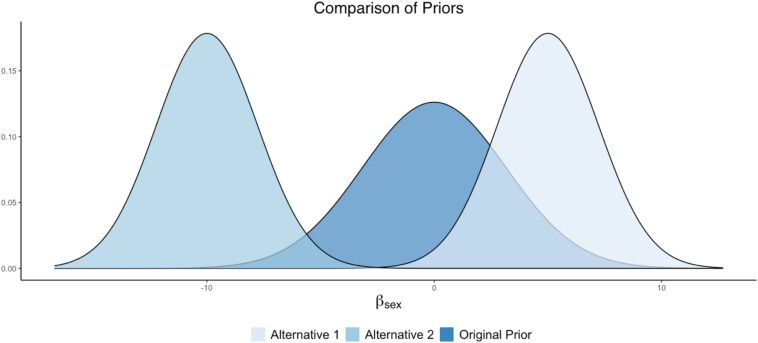
Alternative prior distributions for *Sex* as a predictor of *Cynicism*.

A plot illustrating these prior differences can be found in Figure 3.

#### Parameter 2: *Cynicism* on *Lack of Trust*

For this substantive example predicting cynicism (Figure 1B), we can assume that the researchers based their prior distribution specifications on previous research, indicating that *Lack of Trust* had a strong positive relationship with *Cynicism*. Specifically, assume that the original prior (specified by the researchers) was set at *N*(6, 1), where the value 6 represents the mean hyperparameter (or center) of the distribution and the value 1 represents the variance. This prior density, with a variance hyperparameter of 1, indicates that about 95% of the density falls between 4 and 8. This relatively narrowed prior suggests that the researcher had a relatively strong expectation that a one-point increase in *Lack of Trust* is related to a 4 to 8 point increase in *Cynicism*.

Several competing prior specifications can be imagined for this regression coefficient of *Cynicism* on *Lack of Trust*, each with their own degree of informativeness. The impact of these other prior forms can be examined through a sensitivity analysis. For example, the researcher can examine a diffuse prior distribution, with the intention of downplaying the impact of the prior and emphasizing the data patterns to a larger degree. In this case, a normal distribution can be used as the prior, but the distribution will have a very large spread to coincide with the lack of knowledge surrounding the parameter value. One way of specifying this regression coefficient prior would be as *N*(0, 100). With such a wide variance (akin to Figure 2C), this prior will be largely flat over the parameter space, representing a diffuse prior for this parameter.

Another version of the prior specification can come from an alternative theory on the relationship between *Lack of Trust* and *Cynicism*. Perhaps several experts on the topic of cynicism believe that the degree (or lack) of trust in others has no impact on how cynical a person is. An informative prior centered around zero, with a more narrowed variance compared to the prior described above, reflects this prior belief: *N*(0, 5).

These prior settings would result in an original prior and two alternative specifications such that:

• Original = *N*(6, 1)
• Alternative 1 = *N*(0, 100)
• Alternative 2 = *N*(0, 5).

**FIGURE 4:**
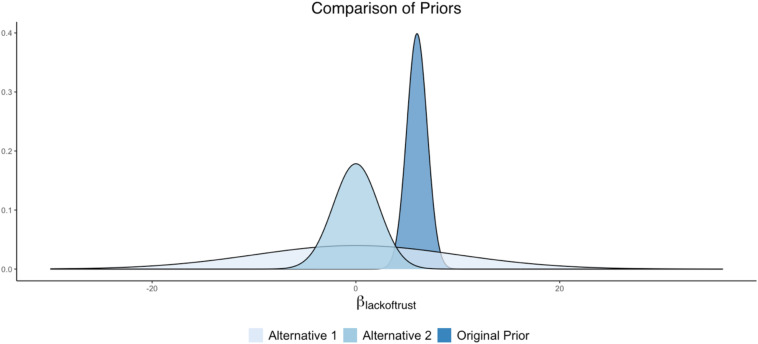
Alternative prior distributions for *Lack of Trust* as a predictor of *Cynicism*.

A plot illustrating these prior differences can be found in Figure 4.

#### Examining Priors for Parameter 1 and Parameter 2 Simultaneously

Finally, the combination of each of these alternative prior specifications can also be compared to examine how prior specifications aligned with alternative theories and previous research impact the posterior results. In total, we can use the App to compare six different models at a time.

### Assessing Convergence

An alternative prior specification can affect the convergence of parameters in the model. As such, model convergence should always be assessed, even if there were no convergence issues with the original prior specification. A converged chain represents an accurate estimate for the true form of the posterior.

**FIGURE 5:**
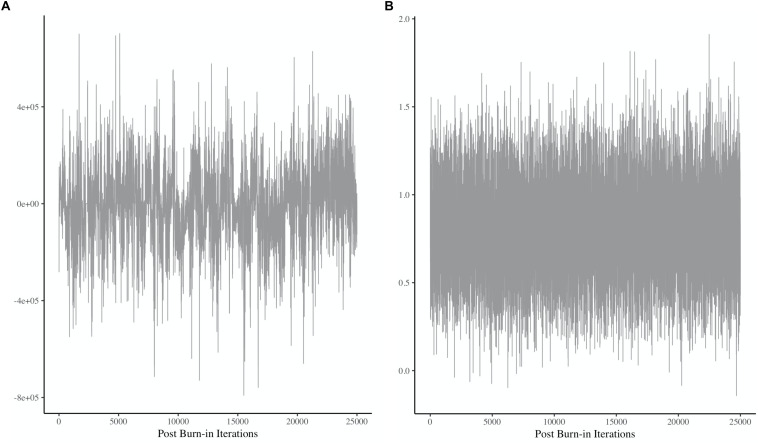
Two chains showing different patterns of (non)convergence. Panel **(A)** shows a great deal of instability throughout the plot, indicating non-convergence. Panel **(B)** shows a relatively stable horizontal mean and variance, indicating convergence. Note that both plots exhibit some degree of autocorrelation, but that is beyond the scope of the current discussion. More information about this issue can be found here: Kruschke (2015) and Depaoli and van de Schoot (2017).

For example, see Figure 5, which presents two different plots showing a chain for a single parameter. Each sample pulled from the posterior represents a dot, and these many dots are then connected by a line, which represents the chain. Obtaining stability, or convergence, within the chain is an important element before results can be interpreted. The mean according to the *y*-axis of Figure 5 represents the mean of the posterior, and the height of the chain represents the amount of variance in the posterior distribution. Convergence is determined by stability in the mean (i.e., horizontal center, according to the *y*-axis) and the variance (i.e., height of the chain). Figure 5A shows that there is a great deal of instability in the mean and the variance of this chain.^9^ The chain does not have a stable, horizontal center, and the height of the chain is inconsistent throughout. In contrast, Figure 5B shows stability in both areas, indicating visually that it appears to have converged. There are statistical tools that can help determine convergence, and they should always accompany visual inspection of plots akin to those in Figure 5. Some statistical tools for assessing convergence include the Geweke convergence diagnostic (Geweke, 1992), and the potential scale reduction factor, or R-hat (Gelman and Rubin, 1992a, b; Gelman, 1996; Brooks and Gelman, 1998).

The beginning portion of the chain is often highly dependent on chain starting values (which may be randomly generated within the software). Therefore, this early portion of the chain is often discarded and referred to as the *burn-in* phase. This part of the chain is not representative of the posterior since it can be unstable and highly dependent on the initial value that got the chain started. Only the *post-burn-in* phase (i.e., the phase of the chain beyond the designated burn-in phase) is considered to construct the estimate of the posterior. The user usually defines the length of the burn-in through some statistical diagnostics, while taking into consideration model complexity [e.g., a simple regression model may require a few hundred iterations in the burn-in, but a mixture (latent class) model may require several hundred thousand]. If convergence is not obtained for a model parameter, then the practitioner can double (or more) the number of iterations to see if the longer chain fixes the issue. If non-convergence still remains, then it may be that the prior is not well suited for the model or likelihood. In the case of a sensitivity analysis, this result could indicate that there is evidence against selecting that particular prior given the current model and likelihood. For more information on convergence and chain length, please see Sinharay (2004) or Depaoli and van de Schoot (2017).

**FIGURE 6:**
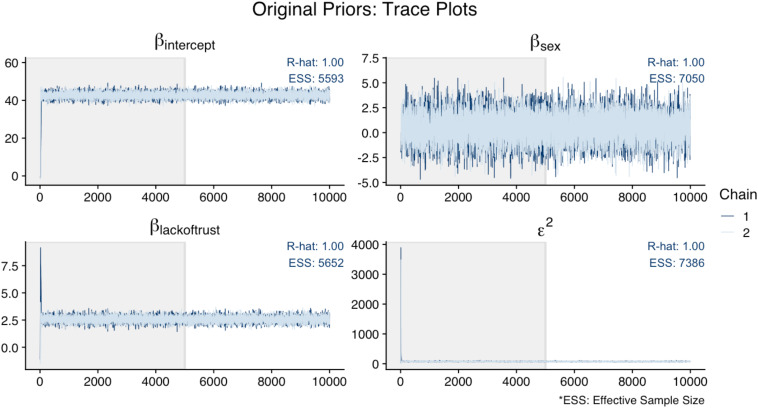
Trace plots of original analysis.

**FIGURE 7:**
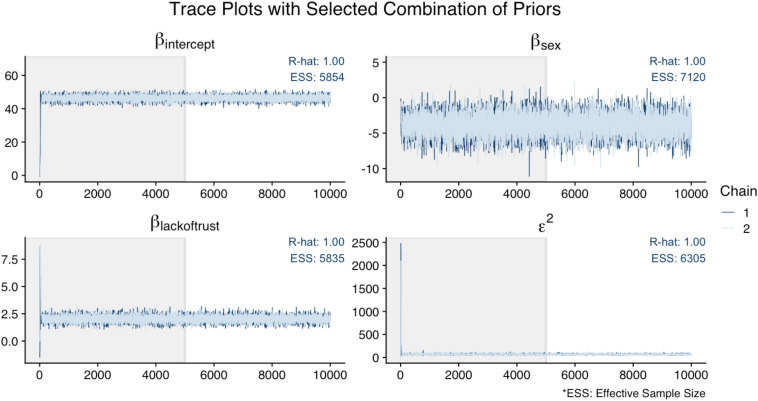
Trace plots of analysis with *N*(–10, 5) prior distribution for *Sex* as a predictor of *Cynicism* and *N*(0, 5) for *Lack of Trust* as a predictor of *Cynicism*.

In the App, we evaluated model convergence visually, using trace plots of the posterior chains, and with diagnostics, using R-hat and the ESS.^10^ Figure 6 illustrates that the trace plots, R-hat (<1.01), and ESS (>1,000) for all parameters in the original analysis indicated convergence. For this illustration, Figure 7 shows the trace plots of an analysis that uses alternative prior specifications for both regression effects: *N*(−10, 5) for *Sex* as a predictor of *Cynicism*, and *N*(0, 5) for *Lack of Trust* as a predictor of *Cynicism*. In this figure, we can see that the trace plot for the effect of *Sex* looks more volatile (though still relatively flat) when using this alternative prior specification; this is most evident by examining the *y-*axis differences across Figures 6, 7. Overall it appears that the alternative priors do not profoundly affect chain convergence, despite some differences with the variance of the chain for the *Cynicism* on *Sex* coefficient (i.e., the variance is wider in Figure 6 for this parameter).

### Inspecting Posterior Density Plots

**FIGURE 8:**
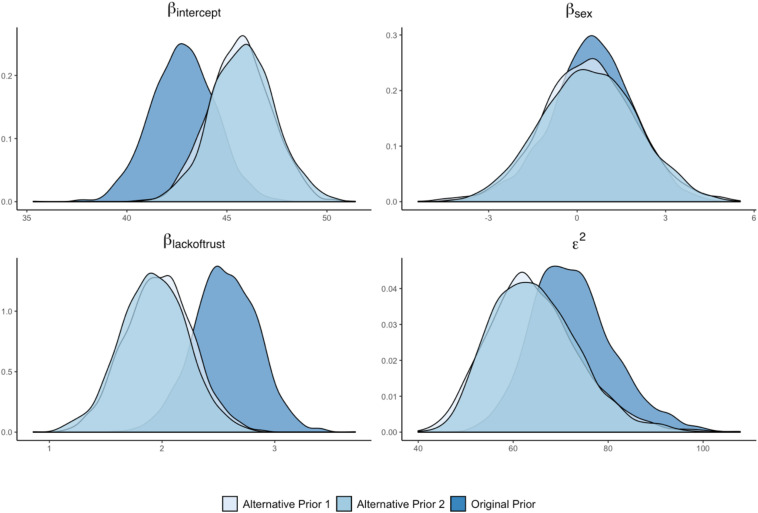
Posterior density plots for original and alternative priors for *Lack of Trust* as a predictor of *Cynicism*.

The next step in the sensitivity analysis is to examine how the alternative prior specifications have affected the posterior distributions of the model parameters. If the posterior distributions are very similar across a range of prior distributions, then it implies that the posterior estimate is robust to different prior distributions. In contrast, if the posterior distribution is drastically altered as a result of an alternative prior, then it shows that the posterior distribution depends more heavily on the specific prior distribution used. For this illustration, we will focus our discussion of the two alternative prior distributions for *Lack of Trust* as a predictor of *Cynicism*. Figure 8 shows that the posterior distribution for the effect of *Lack of Trust* changes as a result of the alternative prior specifications. Both posterior distributions shift to a lower range of values. This result implies that the posterior distribution of the original analysis is affected by the selected prior distribution and that alternative (more diffuse) prior distributions would have resulted in slightly different posterior distributions. In addition, the posterior distribution of the intercept of *Cynicism* shifts to a higher value for both alternative prior distributions, indicating a substantively different definition of the model intercept (i.e., the average value of *Cynicism* when predictors are zero). Finally, the posterior distributions of *Sex* as a predictor of *Cynicism* does not appear to be affected by the alternative priors for the effect of *Lack of Trust*, while the residual variance of *Cynicism* was impacted.

### Comparing the Posterior Estimates

**FIGURE 9:**
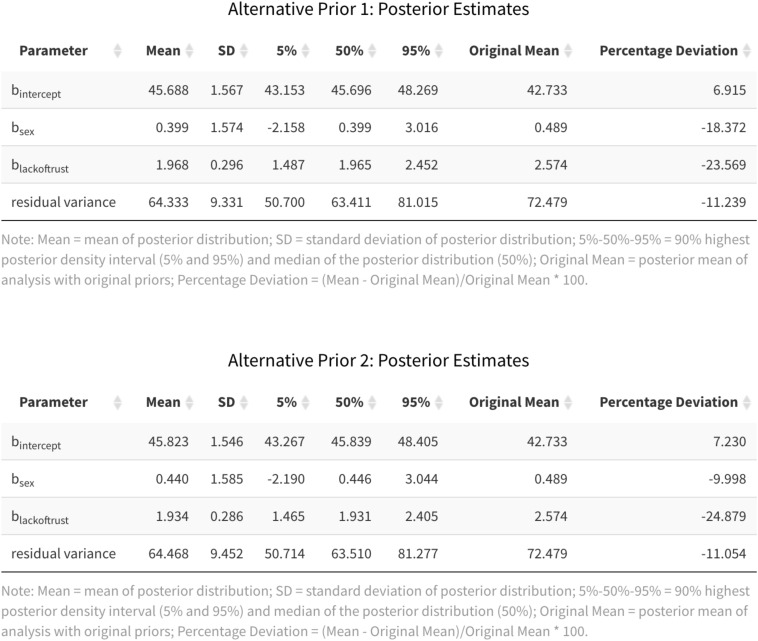
Posterior estimates for the alternative priors for *Lack of Trust* as a predictor of *Cynicism*.

Another way to examine the impact of the prior distribution is to compute the percentage deviation in the average posterior estimate between models with different prior distributions. For this illustration, we will again focus our discussion on the two alternative prior distributions for *Lack of Trust* as a predictor of *Cynicism*. Figure 9 displays summary statistics of the analyses with the alternative prior specifications, as pulled from the App. The final two columns show the average posterior estimates of the original analysis and the percentage deviation between the original and each alternative analysis. In line with the downward shift of the posterior densities of the effect of *Lack of Trust* across the different prior specifications, the percentage deviation is −23.040% or −24.851%, depending on the alternative prior specification. Another way of capturing the impact of the prior distribution is to compare the 90% HPD intervals and see whether the substantive conclusion about the existence of the effect of *Lack of Trust* changes. In this case, zero is always outside the 90% HPD interval, independent of the prior distribution used in the analysis. Thus, the substantive conclusion regarding the role of *Lack of Trust* as a predictor of *Cynicism* does not change across the prior distributions examined here.

### Additional Guidelines for Using the App

We constructed the App so that users cannot examine the combination of different priors in the model before specifying and looking at each one separately. This design-based decision was made for pedagogical reasons. We feel that examining each prior separately is helpful when initially learning about prior impact. The practice of modifying a prior setting and tracking how the posterior changes provides a visual learning experience that enhances discussions surrounding sensitivity analyses. However, in practice, the implementation and variation of priors is more complicated. In the final model being estimated, the combination of priors is the main aspect that matters. There is research highlighting that priors in one location in a model can impact results in another location (see e.g., Depaoli, 2012). Because of this, it is important to examine results with the combination of priors implemented all at once. These results reflect the true impact of the prior settings (as opposed to examining a single parameter at a time). Although this App allows the user to examine one prior at a time (as a learning tool), we note that this may not be a feasible practice in some modeling contexts. For example, some item response theory models have thousands of parameters, and it would only be feasible to examine the combination of priors (rather than one at a time).

The App was designed to enhance pedagogy surrounding visually demonstrating sensitivity analysis. However, we caution the reader that it is indeed the combination of prior settings that drives the substantive impact of the priors.

## Conclusion

Our aim was to present examples (via simulation and application) illustrating the importance of a prior sensitivity analysis. We presented a Shiny App that aids in illustrating some of the important aspects of examining sensitivity analysis results. We have formatted the current section to address frequently asked questions (FAQs) in order to provide an at-a-glance view of the most important components for applied researchers to focus on.

### Frequently Asked Questions About Prior Sensitivity Analysis

(1) Why is a sensitivity analysis important within the Bayesian framework, and what can we learn from it?

A sensitivity analysis is, in many ways, one of the *most important* elements needed to fully understand Bayesian results in an applied research setting. The simulation study, and the demonstration provided in the Shiny App, showed that priors can have a substantial impact on the posterior distribution. Without a sensitivity analysis, it is not possible to disentangle the impact of the prior from the role that the data play in the model estimation phase. A sensitivity analysis can help the researcher understand the influence of the prior compared to the influence of the data. In other words, this analysis can help to establish how much theory [i.e., through informed theory or lack of theory (e.g., diffuse priors)] influences the final model results, and how much the results are driven by patterns in the sample data.

(2) How many different prior conditions should I test during a sensitivity analysis? In other words, how extensive should the sensitivity analysis be?

There is a running saying (or joke) in statistics that the answer to any statistical question is “it depends.” That saying certainly holds true here. In this case, there is no definitive answer to this question, and it really depends on several factors. The extensiveness of the sensitivity analysis will depend on the complexity of the model, the intended role of the priors (e.g., informative versus diffuse), and the substantive question(s) being asked. There are some general guidelines that we can provide. For example, if diffuse priors are implemented in the original analysis, then it will likely not be relevant to include informative priors in the sensitivity analysis. Instead, the practitioner would be better off testing different forms of diffuse priors. However, if informative priors were used in the original analysis, then it would be advised to examine different forms of the informative priors, as well as diffuse prior settings, in the sensitivity analysis. The practitioner must heavily weigh these different aspects and decide on the scope of the sensitivity analysis accordingly. The main goal here is to understand the impact and role that each prior is playing. There are no set rules for achieving this goal since all research scenarios will differ in substantive ways.

(3) What is the best way to display sensitivity analysis results?

Not to borrow too much from the previous FAQ, but the answer to this current question depends on: (1) what the sensitivity analysis results are showing, (2) model complexity—i.e., the number of model parameters, and (3) the number of conditions examined in the sensitivity analysis. In a case where results are relatively similar across a variety of prior conditions, the researcher may opt to have a couple of sentences indicating the scope of the sensitivity analysis and that results were comparable. However, in a case where results are altered when priors differ (e.g., like some of the examples provide in our Shiny App), the researcher may opt for a larger display of results. This could be provided through visuals, akin to the Shiny App plots we presented (e.g., Figures 3, 4, 8, 9), or it may be in a table format indicating the degree of discrepancy in estimates or HPD intervals across parameters. In extreme cases, where there are dozens of parameters crossed with many sensitivity analysis conditions, the researcher may need to put the bulk of the results in an online appendix and just narrate the findings in the manuscript text. Much of this will depend on the degree of the differences observed across the sensitivity analysis, as well as journal space limitations. The important issue is that results must be displayed in some clear fashion (through text, visuals, or tables of results), but what this looks like will depend largely on the nature of the investigation and findings that were obtained.

(4) How should I interpret the sensitivity analysis results?

Sensitivity analysis results are not meant to change or alter the final model results presented. Instead, they are helpful for properly interpreting the impact of the prior settings. This can be valuable for understanding how much influence the priors have, as well as how robust final model estimates are to differences in prior settings—whether they be small or large differences in the priors. Sensitivity analysis results should be reported alongside the final model estimates obtained (i.e., those obtained from the original priors implemented). These results can be used to help bolster the discussion section, as well as make clearer sense of the final estimates. In addition, we discussed an alternative above regarding reporting sensitivity analysis results when diffuse priors are implemented. In this scenario, the practitioner may choose to report results across a range of diffuse priors as the final analysis. This is a strategy that can help illuminate any uncertainty surrounding the exact prior specification if different forms of diffuse priors provide varying results. Finally, if the sensitivity analysis process yields a prior (or set of priors) that produce non-sensical results according to the posterior (e.g., the posterior does not make sense, see Depaoli and van de Schoot, 2017), or results in chains that do not converge, then it may be an indication of a poor prior choice given the model or likelihood. In this case, the prior and results should be described, and it may be useful to describe why this prior setting may not be viable given the poor results that were obtained.

(5) What happens if substantive results differ across prior settings implemented in the sensitivity analysis?

It may initially seem uncomfortable to receive results from the sensitivity analysis that indicate priors have a strong influence on final model estimates. However, this is really not a point of *concern*. Assume sensitivity analysis results indicated that even a slight fluctuation of the prior settings altered the final model results in a meaningful (i.e., substantive) manner. This is an important finding because it may indicate that the exact theory used to drive the specification of the prior (potentially) has a large impact on final model results. Uncovering this finding can help build a deeper understanding about how stable the model (or theory) is. In contrast, if the model results are relatively stable under different prior settings, then this indicates that theory (i.e., the prior) has less of an impact on findings. Either way, the results are interesting and should be fully detailed in the discussion. Understanding the role that priors play will ultimately help lead to more refined and informed theories within the field.

(6) How do I write up results from a sensitivity analysis?

Sensitivity analysis results should be included in the main body of the results section of any applied Bayesian paper. Final model estimates can be reported and interpreted based on the original prior settings implemented. Then the sensitivity analysis can be reported in the context of building a deeper understanding of the impact of the priors. Bayesian results can only be fully understood in the context of the impact of the particular prior settings implemented. After reporting the final model estimates from the original prior settings, a section can be added to the results entitled something like: “Understanding the Impact of the Priors.” In this section, visual or table displays of the sensitivity analysis results should be included. Results of the analysis should be described, and some sense of the robustness (or not!) of results to different prior settings should be addressed. These results can then be further expanded upon in the discussion section, and recommendations can be made about what priors the researcher believes should be further explored in subsequent research. The goal is to provide a thorough treatment of the analysis and give readers ample information in order to assess the role of priors in that particular modeling context.

### Final Thoughts

As we demonstrated through the simulation study and the Shiny App, priors can have a noticeable impact on the final model results obtained. It is imperative that applied researchers examine the extent of this impact thoroughly and display findings in the final analysis report. Visual aids can be a tremendous asset when presenting sensitivity analysis finding, as they quickly point toward the level of (dis)agreement of results across different prior settings.

A key issue when reporting any analysis, but especially one as complicated as a Bayesian analysis, is transparency. It is important to always be clear about what analyses were conducted, how they were conducted, and how results can be interpreted. This issue of transparency is key within any statistical framework, but it is especially an issue for the Bayesian framework because of how *easy* it is to manipulate results by changing prior settings. Bayesian methods are very useful tools, and it is up to us (i.e., the users, publishers, and consumers of research) to set a precedence of transparency and thoroughness when reporting findings. It is our hope that the Shiny App will play a role in promoting the importance of this issue.

## Author Contributions

SD conceptualized and wrote the manuscript. SW and MV made the Shiny App. All authors contributed to the article and approved the submitted version.

## Conflict of Interest

The authors declare that the research was conducted in the absence of any commercial or financial relationships that could be construed as a potential conflict of interest.

## Footnotes

1 Several developments have made this step easier by approximating the posterior instead of estimating it directly (e.g., Gustafson and Wasserman, 1995; Roos et al., 2015). These methods have also been implemented in R packages, such as the ‘adjustr’ package (McCartan, 2020). We do not use this package in our Shiny App as it does not provide the full posterior distribution that we use for our visuals.

2 We do not refer to the traditional sense of the word “bias,” where an estimate is compared to a population value (e.g., in the sense of the comparisons made in the simulation study presented next). Instead, we are referring here to the deviation between two estimates, each obtained as a result of different prior settings. A calculation similar to bias can be implemented, providing the researcher with an indication of the difference between the estimates resulting from the sensitivity analysis. We further illustrate this concept in the section detailing the Shiny App, and we will refer to this concept as “deviation.”

3 The multiple regression model is a simple model and, with the use of conjugate priors (described below), the posterior can be analytically derived without the use of MCMC sampling. However, we felt that using a relatively simple modeling context (opposed to a more complicated, latent variable model, for example) would be useful for describing the estimation elements and other concepts that are illustrated here since these more complicated topics can be generalized to using with complex models that require MCMC.

4 For the purpose of this paper, we will highlight and discuss priors that are normally distributed because they are the most straightforward to illustrate. However, it is important to keep in mind that priors can be specified using a wide range of distributional forms, including distributions that are not named or not proper distributions (e.g., those that do not integrate/sum to 1.0). We discuss other prior forms for non-normally distributed parameters in the App.

5 One could argue that if a prior belief dictated a prior that was not centered at the population value that it would be *correct to the theory*. We use the term “correct” in this simulation study to compare a prior that has been centered over the population value (correct) to one that has been shifted away from the population value though a deviant mean hyperparameter (incorrect).

6 We selected several cells to examine thoroughly for ESS, all had the lowest sample size (*n* = 25) and varying degrees of “incorrect” priors. Most parameters had ESS values of 2,500 or nearby, with some parameters lower. However, all ESS values exceeded 1,800 in our investigation. This amount exceeds the recommendation by Zitzmann and Hecht (2019). Therefore, we believe that the chains in the simulation represent adequate precision. ESS values have also been included in the App, which we describe in the example section below.

7 https://osf.io/eyd4r/

8 Models were estimated through the No-U-Turn sampler (NUTS; Hoffman and Gelman, 2011; Betancourt, 2017) with two chains. Each chain ran for 10,000 iterations, of which 5,000 were discarded as the burn-in.

9 There are many other elements that should be examined regarding the chains, some of which are levels of autocorrelation and the effective sample size. In the interest of space and the goals of the current tutorial, we refer the reader elsewhere to learn more about these topics. Some helpful resources are: Kruschke (2015) and Depaoli and van de Schoot (2017).

10 As mentioned in the section describing the simulation study, the effective sample size (ESS) is directly linked to the degree of dependency (or *autocorrelation*) within each chain. Specifically, the ESS represents the number of independent samples that have the same precision as the total number of autocorrelated samples in the posterior chains. Zitzmann and Hecht (2019) recommend that ESSs over 1,000 are required to ensure that there is enough precision in the chain.

## Appendix

### Prior Sensitivity Analysis Example Write Up

The following section represents a hypothetical write-up of sensitivity analysis results, which mimics the example provided in the Shiny App.

For the first step of our sensitivity analysis, we considered the parameters of most substantive interest in our study. In the case of our regression example, we were particularly interested in the regression coefficients associated with *Sex* as a predictor of *Cynicism* and *Lack of Trust* as a predictor of *Cynicism*. After clearly identifying the parameters of interest, we then identified the most appropriate priors for the original (comparison) priors in the analysis. For example, we selected *N*(0,10) as the prior for *Sex* as a predictor of *Cynicism* and *N*(6,1) as the prior for *Lack of Trust* as a predictor of *Cynicism*. The *N*(0,10) priors suggest *Cynicism* and *Sex* are unrelated, and the *N*(6,1) indicates a positive relationship between *Cynicism* and *Lack of Trust*. In addition to selecting priors for the parameters of substantive interest, we also set *N*(41,10) as prior to the intercept and *IG*(0.5,0.5) as the prior for the residual variance.

To understand the impact of different priors on the posterior distribution, we identified a set of alternative priors to compare to each of our original priors. For our regression example, we selected the alternative priors of *N*(5,5) and *N*(−10,5) for *Sex* predicting *Cynicism*. The *N*(5,5) alternative prior suggests that men have a higher degree of cynicism than women, and the *N*(−10,5) alternative prior means men have a lower degree of cynicism than women. Also, we selected alternative priors *N*(0,100) and *N*(0,5) for *Lack of Trust* predicting *Cynicism*. The *N*(0,100) alternative was much more diffuse than the original prior, suggesting a lack of knowledge about the parameter. The *N*(0,5) has a mean of zero, which indicates no relationship between *Cynicism* and *Lack of Trust*. For the intercept, we selected *N*(0,100) and *N*(20,10) as alternative priors. The *N*(0,100) prior is a diffuse, flat prior, and the *N*(20,10) shits the mean of the original prior downward. Both priors suggest lower cynicism values. For the residual variance, we selected *IG*(1,0.5) and *IG*(0.1,0.1) as alternative priors. The *IG*(1,0.5) is more informative than the original prior, and *IG*(0.1,0.1) is more diffuse than the original prior. Finally, we also specified combinations of these alternative priors to understand the combined impact of different priors on model results.

After selecting our alternative priors, we estimated a series of models with different priors. Each model was checked for convergence via visual inspection of the trace plots, as well as through the R-hat diagnostic. In addition, effective sample sizes (ESSs) were also monitored to ensure that autocorrelation was not problematic. The alternative priors selected yielded adequate model convergence and ESS values. Therefore, we moved to the next step of the sensitivity analysis and inspected the posterior density plots. A visual inspection of the posterior density plots revealed a change in the posterior distributions for *Lack of Trust* predicting *Cynicism* when specifying alternative priors. Specifically, the posterior distribution for *Lack of Trust* predicting *Cynicism* shifts to lower values under both alternative priors, suggesting the prior specification impacts the results. The posterior distribution of the intercept and residual variance of *Cynicism* changed depending on the priors specified, which indicates a substantively different interpretation of the intercept depending on the priors. In contrast, the posterior density plots for *Sex* as a predictor of *Cynicism* were relatively similar, regardless of the alternative prior specification.

We also examined how robust the results were by comparing the posterior estimates across models with different prior specifications. If priors have little impact on the results, then there will be a low percentage of deviation in the posterior estimates between models. However, if the priors have a significant effect, then we will see a higher percentage deviation between models. As expected, given the posterior density plots, we see a downward shift in the estimate for *Lack of Trust* as a predictor of *Cynicism* across different prior specifications. Specifically, the percentage deviation is −23.040% or −24.851%, depending on the alternative prior specification.

Further evidence of the impact of the prior on the posterior distribution can be obtained by comparing the 90% highest posterior density (HPD) intervals. If the substantive conclusions regarding a parameter change depending on the prior, then there is evidence of less robust results. In the case of *Lack of Trust* as a predictor of *Cynicism*, zero is always outside the 90% HPD interval, independent of the prior distribution used in the analysis. Thus, the substantive conclusion regarding the role of *Lack of Trust* as a predictor of *Cynicism* does not change across the prior distributions. This is perhaps the most critical finding of the sensitivity analysis. Although some parameters were more readily impacted in the model by the prior distributions specified, the substantive interpretation of model results did not change depending on the prior specified.”
